# A Clinical Predictive Model of Central Lymph Node Metastases in Papillary Thyroid Carcinoma

**DOI:** 10.3389/fendo.2022.856278

**Published:** 2022-06-16

**Authors:** Zipeng Wang, Qungang Chang, Hanyin Zhang, Gongbo Du, Shuo Li, Yihao Liu, Hanlin Sun, Detao Yin

**Affiliations:** ^1^ Department of Thyroid Surgery, The First Affiliated Hospital of Zhengzhou University, Zhengzhou, China; ^2^ Department of Dermatology, First Affiliated Hospital of Zhengzhou University, Zhengzhou, China; ^3^ Engineering Research Center of Multidisciplinary Diagnosis and Treatment of Thyroid Cancer of Henan Province, Zhengzhou, China; ^4^ Key Medicine Laboratory of Thyroid Cancer of Henan Province, Zhengzhou, China

**Keywords:** papillary thyroid carcinoma (PTC), predictive model, central lymph node metastasis (CLNM), nomogram, risk factors

## Abstract

**Background:**

Thyroid carcinoma is one of the most common endocrine tumors, and papillary thyroid carcinoma (PTC) is the most common pathological type. Current studies have reported that PTC has a strong propensity for central lymph node metastases (CLNMs). Whether to prophylactically dissect the central lymph nodes in PTC remains controversial. This study aimed to explore the risk factors and develop a predictive model of CLNM in PTC.

**Methods:**

A total of 2,554 patients were enrolled in this study. The basic information, laboratory examination, characteristics of cervical ultrasound, genetic test, and pathological diagnosis were collected. The collected data were analyzed by univariate logistic analysis and multivariate logistic analysis. The risk factors were evaluated, and the predictive model was constructed of CLNM.

**Results:**

The multivariate logistic analysis showed that Age (p < 0.001), Gender (p < 0.001), Multifocality (p < 0.001), *BRAF* (p = 0.027), and Tumor size (p < 0.001) were associated with CLNM. The receiver operating characteristic curve (ROC curve) showed high efficiency with an area under the ROC (AUC) of 0.781 in the training group. The calibration curve and the calibration of the model were evaluated. The decision curve analysis (DCA) for the nomogram showed that the nomogram can provide benefits in this study.

**Conclusion:**

The predictive model of CLNM constructed and visualized based on the evaluated risk factors was confirmed to be a practical and convenient tool for clinicians to predict the CLNM in PTC.

## Introduction

Thyroid carcinoma is one of the most popular endocrine tumors, and papillary thyroid carcinoma (PTC) is the most common pathological type (1, 2). The increased incidence of PTC is attributed to both the truly increased prevalence of thyroid diseases and the advances in imaging technology. Current studies have reported that PTC has a strong propensity for CLNM (3), and it is difficult to effectively detect central lymph node metastases (CLNMs) preoperatively (4–6). The most commonly involved central lymph nodes in thyroid carcinoma are the prelaryngeal (Delphian), pretracheal, and the right and left paratracheal nodes; the paratracheal nodes may be anterior as well as posterior to the recurrent laryngeal nerves (3). Whether to prophylactically dissect the central lymph nodes in PTC remains controversial (7). Dissection of the lymph nodes in the central region of the neck is considered necessary. Central lymph node dissection (CLND) is beneficial in eliminating macroscopic or microscopic metastatic sites. When CLNM is found in patients after surgery, a second operation is often necessary. Extra surgery not only is difficult but also increases the risk of complications (8). Prophylactic lymph node dissection of the central cervical facilitates accurate clinical staging (9). However, it has been argued that prophylactic dissection of the central lymph nodes is not necessary. Routine prophylactic CLND is considered uneconomical (10). Routine prophylactic central compartment dissection is particularly associated with temporary and permanent hypoparathyroidism and recurrent laryngeal nerve injury (4, 11, 12). Although numerous retrospective studies determined the benefits of prophylactic CLND by various investigators, the published results have been inconclusive. Due to the indolent nature of PTC, a very large sample size with extended follow-up for a randomized control trial examination is urgently needed. Therefore, preoperative access is of great clinical significance to accurately assess patients for CLNM. In this study, we analyzed the risk factors of CLNM and constructed a predictive model of CLNM to more accurately assess the risk of CLNM preoperatively and provide a practical and convenient tool for clinicians to predict the CLNM in PTC for clinical decision-making.

## Methods

### Study Design

**Figure 1:**
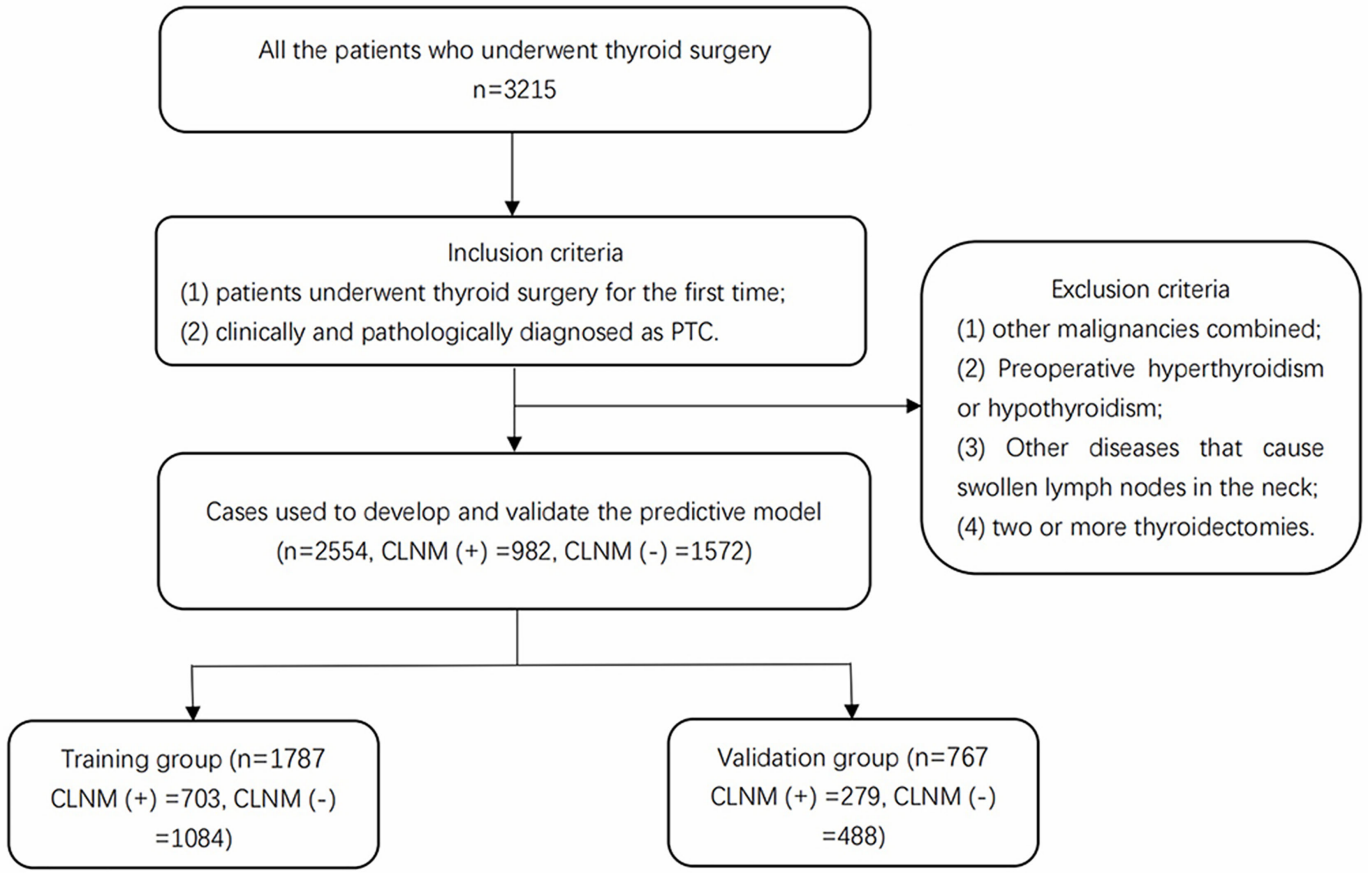
Enrollment flowchart of participants used for model development and validation. PTC, papillary thyroid carcinoma; CLNM, central lymph node metastasis.

We screened all the patients who underwent thyroidectomy for PTC in Thyroid Surgery who were admitted to the First Affiliated Hospital of Zhengzhou University from January 2018 to October 2019. The inclusion criteria were as follows: 1) patients underwent thyroid surgery for the first time; 2) clinically and pathologically diagnosed as PTC. The exclusion criteria included the following: 1) other malignancies combined; 2) preoperative hyperthyroidism or hypothyroidism; 3) other diseases that cause swollen lymph nodes in the neck; 4) two or more thyroidectomies. A total of 2,554 patients including 982 patients with CLNMs (CLNM(+)), and 1,572 patients without CLNMs (CLNM(−)) were enrolled in this study. The enrollment flowchart of the participants is shown in **Figure 1**. The patients with CLNMs (CLNM(+)) and without CLNMs (CLNM(−)) were divided into the training group (n = 1,787) and the validation group (n = 767).

### Data Collection

The basic information, laboratory examination, cervical ultrasound, genetic test, and pathological diagnosis were collected. The basic information included age and gender. The laboratory indices included free triiodothyronine (FT_3_), free tetraiodothyronine (FT_4_), thyroid-stimulating hormone (TSH), thyroid peroxidase antibodies (TPOAb), thyroglobulin antibodies (TgAb), and thyroglobulin (Tg). The characteristics of cervical ultrasound include multifocality and tumor size. Multifocality was defined as more than one lesion observed in cervical ultrasound and pathologically confirmed as PTC. Tumor size was the maximum diameter of the suspected nodule under ultrasound that was pathologically confirmed as PTC (8). All patients had a review of the cervical ultrasound with the same sonographer preoperatively. Genetic test results included *BRAF* and *TERT*. Pathological diagnosis included reports of the paraffin section of the primary lesion and central lymph node.

### Statistical Analysis

Multivariate multiple imputations with chained equations were used to deal with a few missing data of several variables to decrease the bias (13). All the statistical analysis processes involved were completed by R software, version 4.1.1. p-Value <0.05 was considered statistically significant. The classification data were expressed as percentages, and means ± SD or medians (quartile 1, quartile 3) were described as continuous variables that satisfy or do not satisfy the normal distribution, respectively. The odds ratio (OR) values were calculated by univariate logistic regression of the variables. After the collinearity among variables was calculated and the colinear factors were eliminated, the potential variables with a p-value <0.05 were selected to perform the multivariate logistic regression. A clinical predictive model of CLNM in PTC was built based on the variables with statistical senses.

## Results

### Baseline Characteristics of the Variables

The baseline characteristics of CLNM(+) and CLNM(−) are shown in **Table 1**. All the enrolled patients were randomly divided into the training group (n = 1,787) and the validation group (n = 767). There was no significant difference in the levels of these variables between the two groups (**Table 2**). Six potential predictors from 12 candidates were considered to have statistical significance (p < 0.05).

**Table 1:** Baseline characteristics of enrolled patients.

Variables	CLNM (−) (n = 1,572)	CLNM (+) (n = 982)	p-Value
Age [years (IQR)]	47.000 (39.000, 54.000)	41.000 (32.000, 50.000)	<0.0001
Gender, n [male (%)]	269 (17.11)	285 (29.02)	<0.0001
FT_3_ [pmol/L (IQR)]	4.910 (4.530, 5.320)	5.010 (4.630, 5.420)	<0.0001
FT_4_ [pmol/L (IQR)]	11.185 (10.140, 12.322)	11.180 (10.162, 12.190)	0.6288
TSH [μIU/ml (IQR)]	2.530 (1.647, 3.720)	2.504 (1.640, 3.718)	0.3996
TPOAb [(+) (n (%)]	262 (16.67)	170 (17.31)	0.7123
TgAb [(+) (n (%)]	263 (16.73)	190 (19.35)	0.1027
Tg [ng/ml (SD)]	63.154 (404.596)	66.110 (290.648)	0.8422
BRAF [(+) (n (%)]	1,290 (82.06)	841 (85.64)	0.0207
TERT [(+) (n (%)]	4 (0.25)	1 (0.10)	0.6549
Multifocality [n (%)]	308 (19.59)	380 (38.70)	<0.0001
Tumor size (D ≥ 1 cm) (n, %)	382 (24.30)	600 (61.10)	<0.0001

CLNM, central lymph node metastasis; IQR, interquartile range; FT_3_, free triiodothyronine; FT_4_, free tetraiodothyronine; TSH, thyroid-stimulating hormone; TPOAb, thyroid peroxidase antibodies; TgAb, thyroglobulin antibodies; Tg, thyroglobulin.

**Table 2:** Baseline characteristics showed there was no statistical difference between the training group and validation group.

Variables	Training group (n = 1,787)	Validation group (n = 767)	p-Value
Age [years (IQR)]	45.000 (36.000, 52.000)	45.000 (36.000, 52.000)	0.6943
Gender, n [male (%)]	392 (21.94)	162 (21.12)	0.685
FT_3_ [pmol/L (IQR)]	4.950 (4.570, 5.350)	4.950 (4.580, 5.380)	0.8916
FT_4_ [pmol/L (IQR)]	11.160 (10.090, 12.250)	11.260 (10.265, 12.295)	0.2107
TSH [μIU/ml (IQR)]	2.520 (1.660, 3.765)	2.508 (1.630, 3.620)	0.5867
TPOAb [(+) (n (%)]	318 (17.80)	114 (14.86)	0.0794
TgAb [(+) (n (%)]	322 (18.02)	131 (17.08)	0.6078
Tg [(ng/ml, (SD)]	16.100 (7.200, 33.690)	14.700 (6.380, 33.200)	0.2861
BRAF [(+) (n (%)]	1,503 (84.11)	628 (81.88)	0.183
TERT [(+) (n (%)]	4 (0.22)	1 (0.13)	1
Multifocality [n (%)]	482 (26.97)	206 (26.86)	0.991
Tumor size (D ≥ 1 cm) (n, %)	687 (38.44)	295 (38.46)	1

FT_3_, free triiodothyronine; FT_4_, free tetraiodothyronine; TSH, thyroid-stimulating hormone; TPOAb, thyroid peroxidase antibodies; TgAb, thyroglobulin antibodies; Tg, thyroglobulin.

### Risk Factors and Predictive Model of Central Lymph Node Metastasis

**Figure 2:**
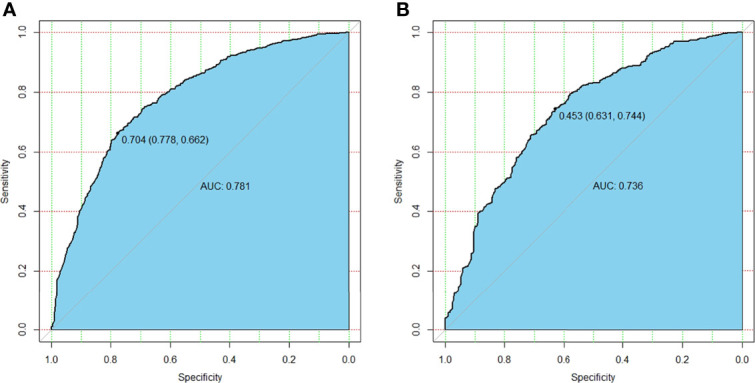
The differential capability of the nomogram. **(A)** ROC curve based on the potential risk factors identified by multivariate logistic regression analysis showed great ability to distinguish the presence or absence of CLNM in the training group with a high value of AUC. **(B)** ROC curve based on the potential risk factors identified by multivariate logistic regression analysis showed great ability to distinguish the presence or absence of CLNM in the validation group. ROC, receiver operating characteristic; CLNM, central lymph node metastasis; AUC, area under the curve.

Multivariate logistic analysis showed that Age (OR 0.947–0.966), Gender (OR 1.541–2.589), Multifocality (OR 2.348–3.815), *BRAF* (OR 1.044–1.926), and Tumor size (OR 4.127–6.416) were associated with CLNM (**Table 3**). The receiver operating characteristic curve (ROC curve) was drawn to evaluate the diagnostic effectiveness of the model (**Figure 2**). The ROC showed a high efficiency with an area under the ROC (AUC) of 0.781 (specificity 0.778, sensitivity 0.662, **Figure 2A**) in the training group. The effectiveness was verified in the validation group with an AUC of 0.736 (specificity 0.631, sensitivity 0.774), and the result is shown in **Figure 2B**. The model showed a great ability to distinguish the presence or absence of CLNM in the training group with a high value of AUC.

**Table 3:** Potential risk factors identified by univariate and multivariate logistic regression analyses.

Variable	Univariable		Multivariable	
	OR (95% CI)	p	OR (95% CI)	p
Age (years)	0.960 (0.952–0.969)	<0.001	0.957 (0.947–0.966)	<0.001
Gender (male, %)	1.948 (1.554–2.445)	<0.001	1.996 (1.541–2.589)	<0.001
FT_3_ (pmol/L)	1.364 (1.158–1.607)	<0.001		
FT_4_ (pmol/L)	0.989 (0.935–1.045)	0.682		
TSH (μIU/ml)	0.990 (0.948–1.031)	0.633		
TPOAb(+) %	1.064 (0.830–1.361)	0.622		
TgAb(+) %	1.232 (0.964–1.572)	0.0935		
Tg (ng/ml)	1.000 (0.999–1.001)	0.349		
BRAF(+) %	1.327 (1.019–1.737)	0.0377	1.414 (1.044–1.926)	0.027
TERT(+) %	0.513 (0.025–4.018)	0.564		
Multifocality %	2.822 (2.278–3.500)	<0.001	2.989 (2.348–3.815)	<0.001
Tumor size (D ≥ 1 cm) %	5.299 (4.313–6.528)	<0.001	5.138 (4.127–6.416)	<0.001

FT_3_, free triiodothyronine; FT_4_, free tetraiodothyronine; TSH, thyroid-stimulating hormone; TPOAb, thyroid peroxidase antibodies; TgAb, thyroglobulin antibodies; Tg, thyroglobulin.

**Figure 3:**
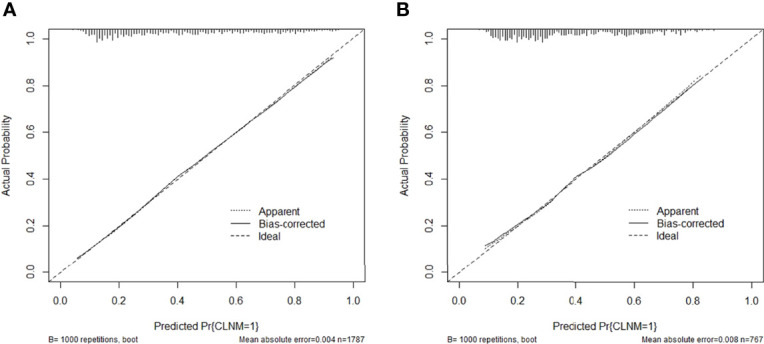
Calibration curve of the predictive nomogram in the **(A)** training group or **(B)** validation group.

Then the model was evaluated by the calibration curve. The predicted values had good consistency in the training group (mean absolute error = 0.004) and the validation group (mean absolute error = 0.008) with the observed variables. The calibration curve showed that the model had a strong calibration ability (**Figure 3**).

**Figure 4:**
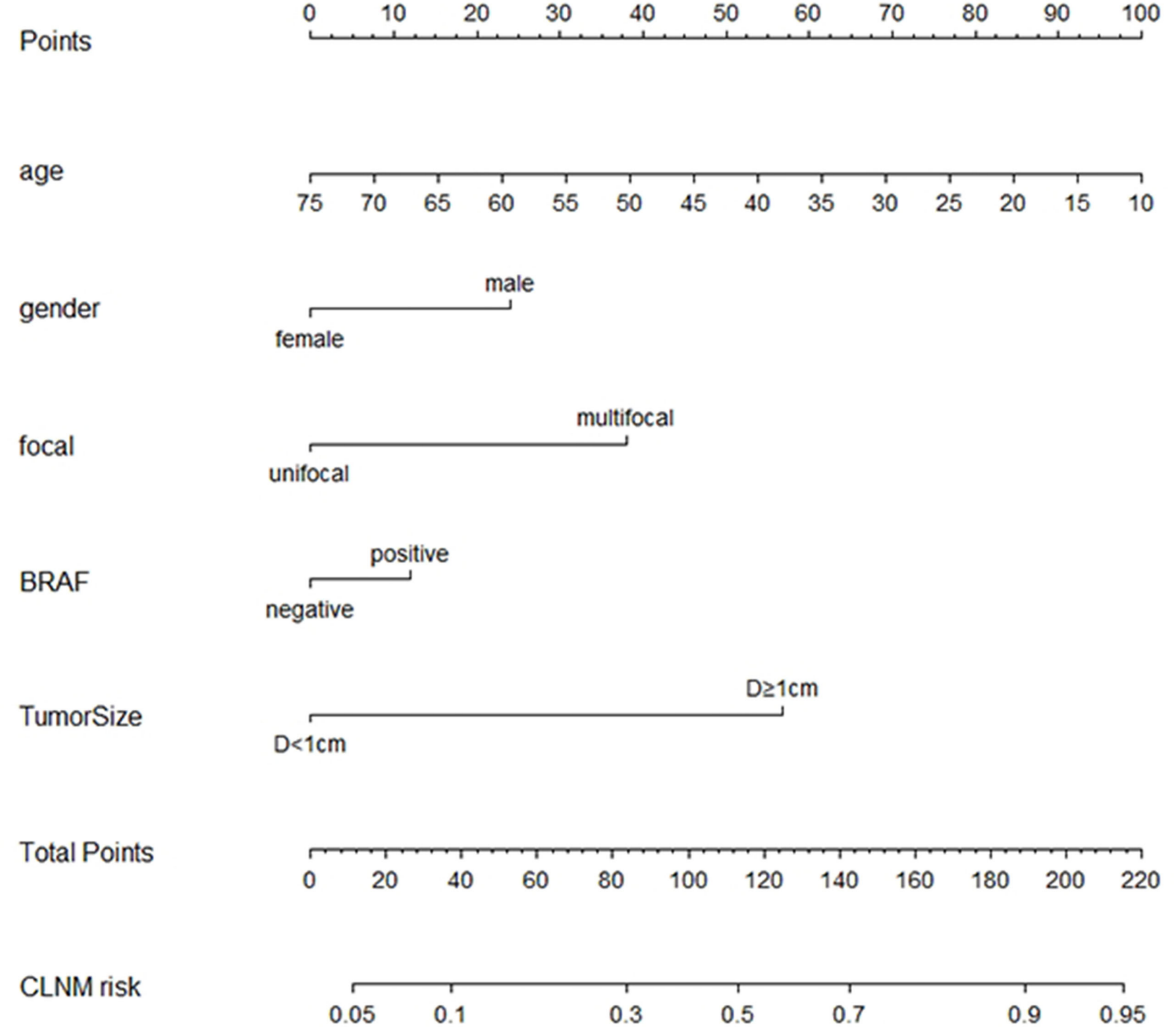
Nomogram based on the five variables including Age, Gender, Focal, BRAF, and Tumor size.

To visualize the model, we plotted the nomogram of our predictive model based on the five variables: Age, Gender, Focal, *BRAF*, and Tumor size. Every variable was scored by drawing a straight line upward the “Points” line. The total points were the sum of the points obtained by the five variables. A straight line down to the axis named “CLNM risk” represents the risk of CLNM (**Figure 4**).

**Figure 5:**
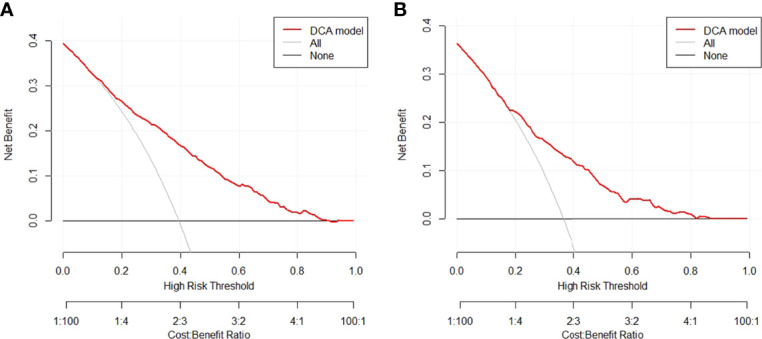
Decision curve for the nomogram predicting CLNM in **(A)** training group or **(B)** validation group. CLNM, central lymph node metastasis.

In the training group, we constructed a decision curve analysis (DCA) to identify the net benefit of the nomogram (**Figure 5**). The curve showed that when the threshold probability of patients is between 0.11 and 0.90, the nomogram can provide benefits.

## Discussion

PTC metastasis occurs most often in the central lymph nodes, with few distant metastases and low mortality, and the rate of cervical lymph node metastasis detection by ultrasound is unsatisfactory, especially for CLNM (5, 14). Information gathered by prospective and randomized clinical studies is the key to determining whether prophylactic dissection of the central lymph nodes in PTC is necessary, but it is probably unavailable currently. Therefore, identification of patients with PTC preoperatively at greater risk of CLNM would be valuable. Prediction models and risk factors analysis based on clinical data have been developed increasingly in a wide variety of diseases in recent years.

To solve these problems, in this study, we collected and analyzed the risk factors and constructed a predictive model of CLNM. The model also showed a high calibration capacity. To the best of our knowledge, it is the first study aiming to construct and visualize a predictive model of CLNM. It is a predictive model that can help clinicians make appropriate treatments and help clinicians assess whether patients need CLND in PTC.

Some studies suggested that CLNM is significantly correlated with age (15, 16), which was similar to our study. Patients with younger age were more likely to be considered at a higher risk of CLNM.

Recent studies have shown that estrogen is a powerful stimulant for benign and malignant thyroid nodules. This explains why thyroid cancer is highly prevalent in women (17). However, in this study, there was a higher rate of CLNM in men. Some scholars confirmed that there were different subtypes of estrogen receptors (ERs) that were considered a protective factor in PTC (17). This may be a potential cause of a higher rate of CLNM in male patients. The detailed knowledge of this regulation in thyroid cancer is still being debated.

Studies have shown that the risk of CLNM increases with the number of foci (11). The multifocal disease was defined as the presence of 2 or more foci of PTC, and each focus was recorded separately. In this study, CLNM rates were high in multifocal PTC with an OR of 2.989 (95% CI 2.348–3.815). With the application of high-resolution ultrasound, the preoperative diagnostic technology of PTC has made a huge breakthrough (18). Therefore, multifocality should be taken seriously during the preoperative ultrasound. When the cervical ultrasound showed that the suspicious lesions have significant multifocality, lobectomy and prophylactic CLNM should be considered.

*BRAF* genetic test was valuable for the diagnosis, prognosis, and therapy of PTC (19). In addition, some scholars revealed that *BRAF* mutations were associated with markers of clinical aggressiveness such as larger tumors, lymph node metastases, and poor clinical outcomes (20). In general, *BRAF* genetic test had strong practicability. Genetic testing in preoperative fine-needle aspiration biopsy (FNAB) was helpful to confirm the diagnosis, patients with a positive result for *BRAF* genetic test should be further evaluated, and there were stronger recommendations for prophylactic CLND.

Tumor size is an important risk factor for CLNM in PTC in the present study. This finding was similar to other studies that the risk of CLNM in PTC increases with tumor size (21, 22). In clinical practice, suspicious nodules larger than 10 mm should be managed with caution. Clinicians need to evaluate the patient’s cervical lymph nodes to decide whether to perform prophylactic dissection of the lymph nodes. Our research, especially the nomogram, provides a good reference for clinicians.

In the univariate logistic regression model, the titer of FT_3_ was statistically significant between the two groups. We searched the relevant literature and found that there is no clear relationship between thyroid hormone and CLNM of PTC. However, one scholar’s research results caught our attention; this study showed a high correlation between PTC microcalcification and thyroid hormones (23). Microcalcification of thyroid nodules indicates that it was more likely to be malignant (24). Therefore, the titer of thyroid hormone may be related to the pathology of thyroid nodules, but it cannot be considered a risk factor for CLNM.

Some scholars have reached similar conclusions (25, 26); younger age, male sex, multifocality, and larger tumor size are the risk factors for cervical lymph node metastases in PTC. The predictive model was also constructed and visualized with a nomogram. However, our study aimed at risk factor analysis and predictive model construction for CLNM with a high AUC. In addition, not only the clinical baseline characteristics but also the genetic test result was included in this study. This was more instructive for patients with preoperative FNAB and *BRAF* genetic test.

The decision curve and nomogram in this study show the great utility of our model, and the 5 items in the nomogram are routine clinical variables that can easily be obtained by clinicians, indicating that it may be beneficial for clinicians to assess the need for prophylactic CLND. Our study has a large sample size of 2,554 patients and has excellent diagnostic effectiveness of CLNM with an AUC of 0.781 in the training group. The operation of the model is simple and fast, which can provide a reference for timely prophylactic CLND.

However, there are also several limitations in our study. First, all the enrolled patients came from the same hospital without external validation. Moreover, we still need to expand the sample size to reduce the heterogeneity. In addition, our predictive model is only suitable for PTC; there is still a lack of predictive ability of our model for other types of thyroid carcinoma.

## Data Availability Statement

The raw data supporting the conclusions of this article will be made available by the authors, without undue reservation.

## Ethics Statement

The studies involving human participants were reviewed and approved by The First Affiliated Hospital of Zhengzhou University Ethics Review Committee. Written informed consent for participation was not required for this study in accordance with the national legislation and the institutional requirements.

## Author Contributions

DY conceived of the idea and provided guidance. ZW wrote the manuscript and completed the figures. QC and HZ contributed to organizing the database. SL, YL, and HS carefully reviewed the manuscript. GD made critical revisions to the manuscript. All authors contributed to the article and approved the submitted version.

## Funding

This study was also funded by the Key Medical Science and Technology Project of Henan Province (SBGJ202101014), which is also from the corresponding author DY, should be placed before funding Major Scientific Research Projects of Traditional Chinese Medicine in Henan Province (No. 20-21ZYZD14).

## Conflict of Interest

The authors declare that the research was conducted in the absence of any commercial or financial relationships that could be construed as a potential conflict of interest.

## Publisher’s Note

All claims expressed in this article are solely those of the authors and do not necessarily represent those of their affiliated organizations, or those of the publisher, the editors and the reviewers. Any product that may be evaluated in this article, or claim that may be made by its manufacturer, is not guaranteed or endorsed by the publisher.
